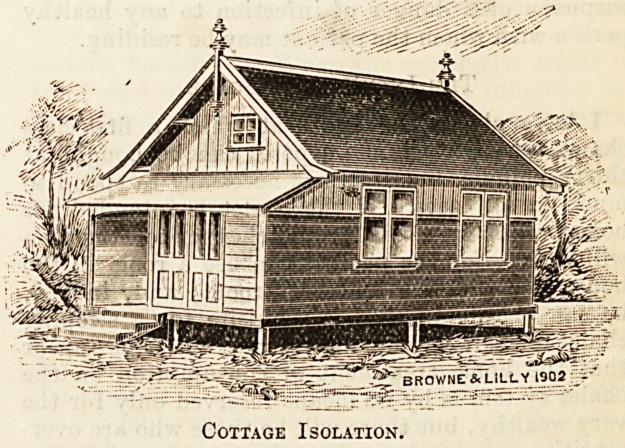# Notes on the Open-Air Treatment of Consumption

**Published:** 1906-10-13

**Authors:** Charles Reinhardt


### Notes on the Open-Air Treatment of Consumption.

By Charles Reinhardt.

When the revolutionary changes which have taken place in our opinions upon the subject of consumption during the last fifty years are considered there is little room for wonder that so much misconception exists amongst the public, especially as to the curability and infectivity of this widespread and fatal disease.

For many centuries consumption was considered, both by physicians and the public, as an almost incurable disorder; those who were afflicted were regarded as doomed to an early and inevitable death, and such treatment as was afforded was directed to the alleviation of symptoms and the promotion of the comfort of the patient rather than to any serious attempt to provide any radical cure of the disease. Thus cough-mixtures were prescribed to relieve the incessant coughing which is so common a symptom, cod-liver oil was administered to check the wasting of the tissues, belladonna was ordered to alleviate the distressing night-sweats, and the patient was confined in close, ill-ventilated, and artificially warmed apartments for fear of "a chill," which, of all things, was to be avoided, as being the commonest cause and most potent aggravator of the disease.

### Early Regulations Against Infection.

As consumption was regarded as being most usually caused by a chill, it was perhaps only natural that its infective character should have been almost entirely overlooked, though it is only fair to our ancestors and predecessors to admit that there were times and places in which the infective character of phthisis was not only recognised, but even exaggerated, in which respect the ancients only anticipated some of our most modern theorists. For instance, in Florence in 1757 an edict was issued in which consumption was described as an infective disorder, and stringent directions as to disinfection were promulgated. Several years earlier the magistrate of Nancy had caused to be publicly cremated in the market-place the goods of a consumptive who had been guilty of sleeping in the same bed with another consumptive. Twenty-five years later an edict was published in Naples by which it was enacted that the clothes of consumptives should be burnt, and that in the event of the recovery of a hospital patient who had suffered from this disease, new clothes should be provided at the public cost; moreover, any person found guilty of selling any clothes formerly worn by a consumptive, and those convicted of knowingly purchasing such garments, should be punished by fine and three years at the galleys.

This edict was not only directed against the mercenary buyers and sellers of infected clothing, for it had a severe penalty in reserve for those who should conceal the nature of the disease from which a consumptive was suffering; indeed, it was enacted that any physician failing to notify a case of pulmonary phthisis should be liable to a fine of three hundred ducats for a first offence, and in the event of a repetition of the misdemeanour he should suffer exile for ten years.

Dr. Alfred Hillier tells of a letter written in 180[illegible] from Rome in which the author, Chateaubriand, complained bitterly that on leaving Rome he was debarred by law from selling his carriages, for which he had counted on receiving two thousand crowns, and added, "Phthisis is declared in Rome a contagious disease, and as Madame de Beaumont (a consumptive) drove two or three times in my carriages, nobody is willing to buy them."

No one would to-day insist upon such drastic rules as those in force amongst the Romans and Neapolitans a century ago; indeed, it is being recognised that there is a tendency on the part of some persons to take an exaggerated view of the alleged dangers of infection from consumptives, as a result of which unnecessary suffering is caused to patients themselves and groundless alarm to the public. It therefore seems desirable that the question of the infectivity of phthisis should be carefully considered, and I propose to return to this subject after dealing with the curability and treatment of the disease.

### Its Infectivity and Extent.

Although, as a result of the general improvement which has taken place during the past half-century in matters of hygiene and in the general comfort and prosperity of the people, the death-rate from consumption has been diminished by no less than 50 per cent., phthisis is still the most important of all diseases in that it kills infinitely more than any other, and it is especially disastrous in that it so often attacks the young adult at an age when he is most productive in labour and when he is likely to have undertaken responsibilities which, if he lose his health, must devolve upon others. It is not desirable that this essay should be burdened with statistics, but it may be mentioned that consumption kills far more than small-pox, typhoid fever, scarlatina, and, in fact, all the infective fevers put together. One death in every six is the result of tuberculosis, and there is no more fruitful source of pauperism and misery, not even excepting the evils of alcoholism.

Now consumption is a preventable disease, and, in its early stages, in the vast majority of cases, curable. Of these facts there is not the shadow of a doubt. In its later or more advanced stages, however, when there has been much destruction of lung tissue, and when inevitable complications have set in, absolute cure is an impossibility, and the chances of substantial and permanent improvement are slight.

When I make the statement that, if taken in the incipient stage, consumption can be cured, I mean that appropriate treatment in an open-air sanatorium, or on open-air lines at home, will result in almost every case in a renewal of good health and working capacity. There will be marked improvement in the general condition, and the local disease will be replaced by a scar; and, if reasonable care be exercised after the period of special treatment at home or in a sanatorium is over, there is no reason why a long and useful life should not be spent. Disappointment and failure seldom follow in cases treated in the earliest stages of the disease, but if left until the disease is advanced, nothing short of a miracle can completely renew the health; and it is only because so many patients have waited until their state was hopeless before entering an open-air sanatorium that there have been a sufficient number of failures to account for the impatience and dissatisfaction of a few thoughtless and inexperienced pessimists.

### Its Cost to the Public.

The Health Commissioner for the City of New York has calculated that consumption costs that city no less than four million six hundred thousand pounds annually; and though no reliable estimate has been compiled in similar fashion with respect to our own metropolis, it is probable that the cost is not far short of six million pounds in London alone. We know that in England and Wales sixty thousand deaths occur from tuberculosis each year, and that the majority are amongst the wage-earning adults of the working classes, and that, as a consequence, an immense number of helpless children and widows are thrown upon the support of the rates. It is obvious that, whether directly or indirectly, the expense and loss sooner or later must fall upon the public purse, and therefore, if only from a business point of view, it is well to examine the facts closely to discover the most economical remedy.

And yet our organisation for the treatment of the consumptive poor is extremely unsatisfactory, and it must be remembered that consumption is a disease which, under existing conditions, spreads from the poor to the wealthier classes, who otherwise, even with very moderate precautions, would be practically immune.

### Present-day Treatment a Mockery and a Scandal.

Dr. Heron speaking recently, in the course of a discussion at the Royal Sanitary Institute, London, on the subject of the Open-air Sanatoria, described the treatment habitually accorded in London to the consumptive poor as a "mockery and a scandal."

Let us take as a typical instance the case of a workman earning, say, two pounds a week, and living with his wife and children in modest comfort. He has a good home, neatly furnished, and he is devoted to his work and to his domestic joys. He is honest, industrious, and ambitious, and, indeed, given good health, is almost an ideal citizen; but one day he realises that there is something wrong. He has felt tired and languid of late, and has never quite shaken off the cough left by last winter's sharp attack of influenza; moreover, he is getting thin; when he hurries or walks up hill he soon gets short of breath. So he decides to see the club doctor, who examines his chest and tells him that he ought to get an "out-patient letter" for one of the chest hospitals. After a few days, therefore, he is to be found waiting amongst a crowd of similar unfortunates in the out-patient department of, let us say, the Brompton Hospital, in Fulham. By this time he is seriously alarmed, though ignorance still shields him from the keener suffering yet in store for him. At length his turn comes, and he is ushered into the presence of a physician whose advice he has come to obtain. He is questioned, and examined physically, and a bacteriological test of his expectoration is made, and he is informed that he is suffering from pulmonary tuberculosis. A piece of blue paper and a quart bottle of medicine are given to him, and he is told to come again in a fortnight.

### A Sad Picture.

He returns home dejected, if not desperate, and, seated in the arm-chair in his living room, he takes the blue paper from his pocket and reads it. He is told therein that he is consumptive, and that he is a danger to himself, to his wife and family, and also to his fellow-workmen. He has good reason for the profound depression which overwhelms him, though perhaps he only half guesses the dismal truth that he is a doomed man, and that the large bottle of tonic expectorant mixture is practically useless, and certainly incapable of arresting the progress of the fell disease which has attacked him. He, perhaps, only realises as a dim possibility that his working powers will soon fail him, that his companions at the workshop will be afraid of him, and that he will, before long, lose his situation altogether, and be dependent upon his scanty savings and the temporary sick pay from his club, supplemented, perhaps, for a while by the assistance of relations or gifts from charitable persons.

After a few months we find him still at work, but his cough is worse than before, and he is weak, emaciated, and hollow-eyed. He still makes a visit once a fortnight to the hospital, and carries away a quart of useless medicine and an imperfect recollection of wholly impracticable advice: "You must feed up, my good man," says the physician, "take plenty of good nourishing food, and live night and day in the open air; avoid sudden exertion, and don't worry." It would be just as sensible to tell him to go for a cruise in his yacht, or to take a trained nurse and a valet and spend the winter amongst the Alps. The physician knows all the time that the treatment actually offered and the medicine given are a delusion and a farce, or, indeed, in Dr. Heron's words, "a mockery and a scandal."

### The Descent to Poverty.

A little later we find the patient bed-ridden and helpless, occupying the room shared at night, and perhaps by day as well, by his wife and children. By this time extreme poverty has transformed the once neat and comfortable home into an ill-furnished, ill-kept, and comfortless place, which has none of the characteristics which we associate with the word "home." Very soon it becomes impossible to pay the rent, and the family find themselves the occupants of a single room in a cheap tenement; and now, if not before, each and every member is running grave risk of contracting the disease, and the number of consumptives is likely to be increased, to the real detriment of the community at large.

### Gratuitous Sanatoria Tried.

In the meanwhile, strenuous efforts have doubtless been made on behalf of the patient and his family by well-meaning and kindly persons. His former employer has seen that his name has been put down for admission to one of the few gratuitous open-air sanatoria; but as there are a hundred or more names before his, and as the accommodation is very limited, for reasons which will presently appear, it is probable, if not certain, that he will be dead or too far advanced in the disease for cure to be possible by the time his turn for admission arrives. A charitable and philanthropic lady has interested herself in the case, and after considerable correspondence has ascertained that admission to the Royal Hospital at Ventnor can be obtained by means of a subscriber's " letter " in the course of a month or two, at a payment of ten shillings a week, or that it might be possible, after some delay, to get him into the City of London Hospital for Diseases of the Chest, but that in this case there would be a strict limit of a very few weeks, after which he would be discharged to find treatment elsewhere, so that the chances of substantial benefit would be so slight as to make it hardly worth while to compete for the next vacancy, and to undertake his removal when admission was secured. Similar difficulties are found to exist in the case of Mount Vernon Hospital, at Hampstead, with its country sanatorium at Northwood, and at the Victoria Park Chest Hospital; and the London Open-Air Sanatorium at Wokingham is found to be out of the question, though built for the National Association for the Prevention of Tuberculosis, to which it was presented as a free gift by a millionaire philanthropist, and presumably a charitable institution, a charge of three guineas weekly is made for each patient. And so nothing is done and at last the end comes, and the patient is received in a pauper's grave, his unhappy family becoming a charge upon the rates. His widow, broken in health and infected with tuberculosis, soon follows her ill-fated husband along the dismal road leading through the wards of the workhouse infirmary to a nameless grave in a Poor-law cemetery. And so two lives, at least, have been wasted, and the community has been robbed of the services of two useful adults and has become responsible for the maintenance of a young family, more than one member of which is probably already infected with tuberculosis. And yet the whole of this tragedy might have been averted, as ten thousand similar ones might be if only a little intelligence were directed to a problem which is one of the most pressing questions of the day and yet susceptible of a ready and an easy solution.

### The German Method.

A healthy, sober, and industrious man is surely worth £200 to the community. His untimely death doubtless costs a larger sum, since the support of a young family has often to be met; and I therefore contend that it would pay well to provide early and efficient treatment for incipient consumptives, and that this is of importance only second to prevention of the disease. If, however, my contention seems too theoretical, I would urge in its favour that the assurance societies in Germany have demonstrated that it pays better to erect and maintain open-air sanatoria and to treat incipient consumptives than to meet the claims at their death and provide the pensions for their widows and families. The view taken by the German assurance societies may be explained, in a business-like fashion, as follows: A man of moderate means may be expected to insure his life for £200—the sum at which, in a very modest estimate, I may assume the money value of an adult citizen to the State. It must be remembered that in Germany adult male members of the industrial classes are compelled to insure their lives, a deduction from their wages being made for the purpose of paying the premiums. In the event of the death of such a man from consumption the insurance society will have to pay £200 to his relations. If, on the other hand, the disease can be arrested in its early stages, the workman, the insurance company, and the State will all be gainers. It costs about 20 marks, or £1 sterling, for each week of treatment, and the average term for incipient cases is four months; but to allow for losses in respect of incurable and exceptional cases let us allow six months, or twenty-six weeks, as the average period. It therefore comes to this—that the German assurance societies prefer to pay from £17 to £26 to restore a man to health, than to allow him to die of consumption for want of sanatorium treatment, in which event the loss of his life will cost them a much more substantial sum.

Surely if it pays the German companies, who have nothing beyond their finance to move them, it would be more than worth while in England to save incipient consumptives by the provision of sanatoria and by educating the people as to the advantage of the open-air methods, when by so doing we are not only saving life and wealth, but at the same time hastening on the final extinction of tuberculosis.

### Sanatoria for the Poor.

It has doubtless long been recognised that it would be a very good thing to provide open-air sanatoria for the poor, and steps have been taken to this end by the authorities of certain hospitals, by a number of county councils, and by various Local Government bodies, encouraged by the example of the King himself, who devoted the sum of £200,000, given him for charitable use nearly five years ago by Sir Ernest Cassel, to the erection of the King Edward VII. Sanatorium, near Midhurst—a costly and substantial edifice which is only just ready for the reception of patients, and which when full will only accommodate a hundred patients, of which eighty-eight will be required to pay two guineas weekly, the remaining twelve being charged no less than six and eight guineas a week. The real difficutly, however, has been, and remains, that of finance; and if the King's Sanatorium and several others recently erected at Frimley, Northwood, and elsewhere are taken as examples of what is necessary in the way of structure and expense, there is no wonder that the public authorities and the charitable public have shirked their responsibilities and missed their opportunities in the matter, since there has been a colossal waste—ten times the necessary cost having been squandered on palatial edifices that are not only unnecessary but positively disadvantageous so far as efficiency is concerned.

The best open-air sanatorium consists of a number of separate sleeping chalets, each for one patient only, surrounding an administrative building containing dining-room, kitchen, domestic offices, and staff quarters. Each chalet should have floor space ten feet square, and two windows opening in each of four directions; it should be raised on blocks of wood or stanchions to permit of air circulation beneath the floor, which keeps the latter dry and prevents rot. A verandah, four feet in depth, should face the south-west, and upon this the patient can sit or recline in preference to congregating with other patients in a common verandah or "leigehalle." The cost is £30, and the cost of the administrative block, including furniture and equipment for the whole institution, and even the freehold of the ground within fifty miles of a large town such as London works out at less than an additional £70 per bed; so that the best and most efficient open-air sanatorium can be provided at a total outlay of less than £100 per patient. The King's Sanatorium at Midhurst cost upwards of twelve times this sum; and, indeed, the interest on the capital outlay at 5 per cent. would actually provide the whole cost of food and upkeep and render an institution for the same number of patients independent and self-supporting. These statements are not amateur theories, but are the result of prolonged practical experience, of which the following is an item and an instance.

### The Chalet System.

Several years ago I was approached by a charity committee which had collected £1,000 for the erection of an open-air sanatorium for necessitous patients. I built eight chalets at £30 each and a seven-roomed administrative block for £300. The equipment of furniture, linen, etc., cost £160, and the value of the ground, with good shelter and mature trees affording shade, and an ample supply of pure water available from a water company's main is about £40 an acre. An advantage of the chalet system is its elasticity; for single chalets can easily be added, and, contrary to the expectations of inexperienced theorists, there is no especial difficulty in managing a large chalet sanatorium. It may also be mentioned that the Open Air League, of which I have the honour to be the Honorary Secretary, and which includes upon its Committee Drs. James Goodhart, George Heron, Percy Kidd, Wilfred Hadley, Vaughan Harley, etc., is opening a sanatorium near Clacton, wherein twenty-five patients are to be treated at an inclusive estimate cost of 25s. a week per bed.

A Garden Chalet.

### Criticism of the Method.

Sceptics are not wanting who assert that a chalet sanatorium is unsatisfactory for at least three reasons—namely, difficulty of service, discomfort to patients, and the perishable character of the buildings. But these objections are easily answered. A chalet sanatorium for well-to-do patients certainly involves extra cost for service, amounting, in my experience, to not more than 15 per cent. on the amount allotted for servants, nurses, and attendants; but this does not apply to a chalet sanatorium for poor patients in the early stages of the disease, who are quite able to wait upon themselves for the most part, and need little nursing or domestic service. There is much less discomfort in a chalet sanatorium than in a large hospital-like institution. In the latter there are inevitable

Chalet Sanatorium.

chilly floor currents of air at times, which are absent in the case of a chalet. Patients who have occupied a properly constructed separate sleeping chalet regret the return to a room on leaving the sanatorium, and many who can afford it erect one in their own garden on returning home. In a word, the chalet is better and more comfortable than the best of rooms in a large building, which latter, unless in a corner position, can only have one aspect, as against four in the case of a chalet.

### Chalets on the Chiltern Hills.

Temporary buildings, if chalets may be so termed, are best for the open-air treatment, for bricks and mortar are unnecessary. Chalets which have been in use on the Chiltern Hills for the past six years are now in perfect repair, and the only cost has been represented by a coating of paint, which has not even been vouchsafed in every case. It must be remembered that the fact that the chalets are raised on stanchions secures them from rotting and damp, which shortens the lives of wooden buildings even if on a brick foundation but without provision for a free current of air beneath the floors.

At the debate referred to earlier in this article one speaker stated that he was in entire agreement with the contention which I urged respecting the chalet sanatoria so far as small institutions were concerned, but that the idea was impracticable for large sanatoria. Nothing could be less justifiable than such a statement. I have yet to learn that anyone having the requisite experience endorses such a view. During the past six years I have been constantly adding new chalets at the sanatorium on the Chiltern Hills already mentioned, and I have not observed that the increase in number has led to any increase in the difficulties of administration. I have, indeed, found that extra numbers decrease the proportionate cost and trouble both in sanatoria on the chalet principle and in the case of sanatoria consisting of large self-contained buildings, with the management of which I have also been concerned.

### Some of the Results.

The results obtained amongst patients occupying chalets are, in my experience, 20 per cent. better than in the case of patients treated in rooms in a large building; hæmoptysis, or bleeding from the lungs, is reduced to a minimum, night sweats are practically obviated, draughts are replaced by pleasant breezes or avoided by simply closing the windows on the exposed aspect, leaving the remainder widely open, and, finally, more rapid and satisfactory progress is made. Unfortunately, however, the minds of those who are responsible for hospital construction or who have the opportunity of erecting sanatoria appear to be positively obsessed with the love of bricks and mortar, and the obsession results in a pitiful waste of human life.

I have suggested that an adult belonging to the industrial class is worth £200 to the community, and that his death from a preventable disease involves the loss of at least this sum. Now £60 would provide treatment in a sanatorium for a whole year, which in incipient cases would seldom be necessary, and £5 per patient would represent the interest on the capital outlay incurred in the provision of an adequate number of chalet sanatoria, and a little extra expense would provide some amount of outdoor relief to the family during the husband's residence in a sanatorium and secure the patient training in an open-air industrial colony to fit him for more suitable work than that in the performance of which he lost his health. But in making these suggestions I am going much farther than the actual necessities of the case require or than present opportunities offer. The essential points which I wish to press home are that the best and most efficient open-air sanatorium can be provided, including the

Cottage Isolation.

freehold of the land, for £100 a bed, and that a patient can be maintained at the rate of £60 a year, and that the County Councils, Boards of Guardians, or Local Sanitary Authorities are already empowered by law to provide sanatoria; though out of 700 councils in England and Wales 600 have failed to take any steps in this matter, doubtless owing to false conceptions as to the expense involved.

The Public Health Act, 1875, and the Isolation Hospital Act, 1893, confer powers on County Councils which would enable them to provide sanatoria for the poor, and, if necessary, to borrow money for the purpose.

### Equally Beneficial to the Rich.

So much then for the treatment of the consumptive poor, though it will be clear that the chalet sanatorium is equally beneficial and appropriate for rich and poor; but in the case of the well-to-do a sanatorium is not by any means a necessity. I am aware that there are advantages in sanatorium routine, in the supervision of the physician-in-charge, and in the removal from domestic worries and irritations which residence in a sanatorium away from home secures; but I have satisfied myself by repeated observation that consumptives who have means and opportunities to enable them to carry out the open-air treatment at home or in the comparative privacy of a cottage with a good garden are generally independent of such a semi-public institution as a sanatorium. Indeed, I know that in not a few cases there have been advantages in the home treatment which quite outweighed those of the sanatorium. To such the chalet system is indeed a boon, since it enables them to convert a country cottage or their own residence into an efficient and private sanatorium with little cost or inconvenience; and under such circumstances there need be no separation from wife or family and no suspicion of a danger of infection to any healthy person with whom the patient may be residing.

### The Infectivity Scare.

I have placed the figure at which a first-class chalet for sanatorium purposes can be bought at thirty pounds, but it is not necessary to spend so much in all cases. If the chalet is intended for home treatment a smaller and cheaper construction will suffice, for the patient can use a room in the house is a dressing and bath-room; and, indeed, he can obtain all that is requisite for a sleeping chalet for half the sum named. It will thus appear that the home treatment of consumption on the chalet system is by no means reserved only for the very wealthy, but there will be those who are over-sensitive or misinformed on the question of the infectivity of consumption who will raise the cry that in all cases the phthisical patient should be sent away from home to a sanatorium, if not altogether for his own benefit, at least for the safety of his family and friends. And this brings me again to the infection scare. That such a scare exists even amongst cultured and educated people is unfortunately only too true. I knew a case in which a patient who had been restored to comparative health in a sanatorium, and who, so far as bacteriological examination could reveal, had been free from tubercular bacilli for several years, on returning home was received with such evident and undisguised nervousness and anxiety by her family that she only longed to return to the sanatorium, or to go anywhere to escape from treatment which might, perhaps, without excuse, have been accorded to a leper. It is true that in the technical sense phthisis is an infectious disease, and, indeed, that every case is derived from an antecedent one, but it is equally certain that the infection is almost always conveyed from one patient to another by means of the sputum, which, when properly collected and promptly destroyed, as always happens in an open-air sanatorium, or in a home where a patient is being properly treated, there is no real danger of infection to the healthy inhabitants, either of a sanatorium or private household. Indeed, there is less danger in living ten years in a sanatorium, properly conducted, or in a house in the grounds of which a patient is being treated in a properly constructed chalet, than there is in spending a month in an ordinary hotel, or in frequenting railway trains, theatres, churches, and other public buildings or closed places of public resort during a single week, for the simple reason that under the first set of conditions there is little likelihood of meeting the tubercle bacillus in the atmosphere, whilst in the public places afterwards referred to it is almost certain to be met with practically at all times. In fact, if one's object in life is to avoid the infection of consumption—and this seems to be so in not a few cases—the safest place in the world is an open-air sanatorium or a household where, owing to the presence of a patient undergoing the open-air treatment, at the hands of a physician, such simple and sensible precautions are taken as will ensure the purity of the atmosphere so far as the tubercle bacillus is concerned.

I have devoted this article to the treatment of consumption by means of open-air methods on the chalet principle because it is well known and beyond dispute that consumption is preventable and curable by the methods indicated. I have purposely remained silent as to the methods of treatment involving the use of sera, as the benefits of such methods, whether on the lines of Dr. Koch's tuberculin or any modification thereof, or of Professor Sir Almroth Wright's opsonic system, or of Professor Behring's new serum, are as yet problematical.

## Figures and Tables

**Figure f1:**
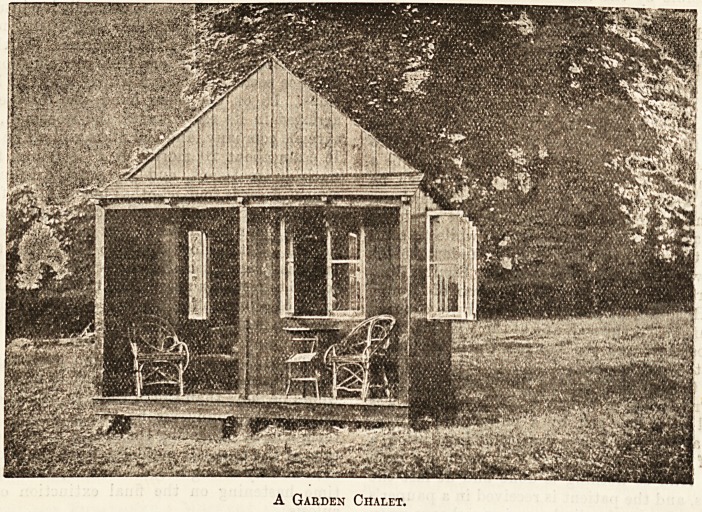


**Figure f2:**
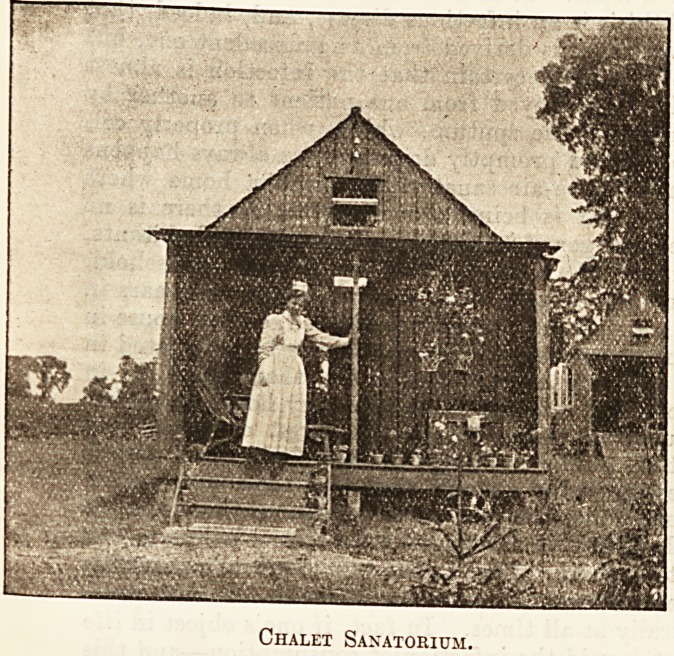


**Figure f3:**